# Origin of ammoniated phyllosilicates on dwarf planet Ceres and asteroids

**DOI:** 10.1038/s41467-021-23011-4

**Published:** 2021-05-11

**Authors:** Santosh K. Singh, Alexandre Bergantini, Cheng Zhu, Marco Ferrari, Maria Cristina De Sanctis, Simone De Angelis, Ralf I. Kaiser

**Affiliations:** 1 Department of Chemistry, University of Hawaii, Honolulu, HI USA; 2 W. M. Keck Research Laboratory in Astrochemistry, University of Hawaii, Honolulu, HI USA; 3 Istituto di Astrofisica e Planetologia Spaziali, INAF, Roma, Italy; 4 Present Address: Federal Center for Technological Education Celso Suckow da Fonseca, Rio de Janeiro, Brazil

**Keywords:** Geochemistry, Physical chemistry

## Abstract

The surface mineralogy of dwarf planet Ceres is rich in ammonium (NH_4_^+^) bearing phyllosilicates. However, the origin and formation mechanisms of ammoniated phyllosilicates on Ceres’s surface are still elusive. Here we report on laboratory simulation experiments under astrophysical conditions mimicking Ceres’ physical and chemical environments with the goal to better understand the source of ammoniated minerals on Ceres’ surface. We observe that thermally driven proton exchange reactions between phyllosilicates and ammonia (NH_3_) could trigger at low temperature leading to the genesis of ammoniated-minerals. Our study revealed the thermal (300 K) and radiation stability of ammoniated-phyllosilicates over a timescale of at least some 500 million years. The present experimental investigations corroborate the possibility that Ceres formed at a location where ammonia ices on the surface would have been stable. However, the possibility of Ceres’ origin near to its current location by accreting ammonia-rich material cannot be excluded.

## Abstract (web summary)

The authors here propose a chemical reaction that forms ammoniated phyllosilicates on Ceres. This process could trigger at a very low temperature, suggesting Ceres evolution in a region different from its current location.

## Introduction

The Dawn spacecraft and ground-based telescopes captured spectroscopic signatures of ammoniated minerals on the surface of dwarf planet Ceres^1–5^. The observed infrared features at 3.05 µm (3278 cm^−1^) and 3.10 µm (3225 cm^−1^)^4,6,7^ are consistent with ammoniated (NH_4_^+^) phyllosilicates such as NH_4_^+^-montmorillonite and NH_4_^+^-annite^3,8^. The widespread occurrence of ammoniated phyllosilicates^6,9,10^, is inextricably linked to the planetary evolution history of the dwarf planet. However, understanding the origin and formation mechanisms of these minerals on Ceres is yet elusive. The ammoniated minerals on Ceres’ surface could have originated from the reaction of clay minerals with ammonia (NH_3_), present in the form of ice and/or ammonia-bearing organic matter^3,11^. These processes may have been triggered by thermal alteration and/or space weathering through the exposure of Ceres’ surface to the Solar Wind and Galactic Cosmic Rays over geological time scales^3,4^. Very recently, the formation of ammonium salts at the surface of comets has been proposed to occur via acid-base reactions propelled by thermal processing of the ices^12^. Although the surface compositions of comets and those of Ceres are distinct, presumably similar thermal processing could initiate proton transfer reactions possibly along with nucleophilic addition reaction(s) between ammonia and phyllosilicates clays during the evolution of Ceres. Intriguingly, space weathering could also facilitate coalition of ammonia with minerals to form ammonium ions^13^. However, the lack of fundamental, low temperature laboratory experiments on the transformation of ammonia-coated minerals to ammoniated silicates under realistic Solar System conditions, leaves the question of the source of the spectroscopic signatures of ammoniated minerals on Ceres’ surface open.

In this work, we perform laboratory simulation experiments to test the hypothesis of a low-temperature triggered formation of ammoniated montmorillonites through proton transfer processes from montmorillonite—a phyllosilicate similar to the ones composing Ceres’ regolith—^14^ to ammonia under realistic astrophysical conditions representing Ceres’ surface. Our investigation shows that the proton exchange reaction between phyllosilicates and ammonia (NH_3_) is a facile pathway to the formation of ammoniated phyllosilicates, which could initiate at temperature as low as 54 K. Furthermore, we reveal the thermal (300 K) and radiation stability of ammoniated phyllosilicates over a timescale of 500 Million years. The present study reports the first experimental observations, which validate the possibility of Ceres evolution at a location where ammonia ice on the surface would have been stable.

## Results

### Experimental scheme

The experiments were conducted in an ultra-high vacuum (UHV) chamber to eliminate any surface contaminations (Supplementary Fig. 1)^15,16^. Two distinct sources of montmorillonite were exploited for the experiments: (i) a commercial montmorillonite and (ii) a naturally occurring montmorillonite^8^. Montmorillonite samples from two different origins were considered to investigate the effect of mineral’s composition on the ammoniation process. Powdered montmorillonite samples were pressed onto a polished silver substrate, which was interfaced to a low temperature cryostat, to form layers with thicknesses of 80 ± 10 µm. Following surface science cleaning techniques, the mineral samples were first cooled to a temperature of 5.0 ± 0.5 K. Ammonia gas was then deposited onto the cooled mineral layers to form ammonia ice with a thickness of 600 ± 30 nm (Supplementary Fig. 2 and Note 1). These samples are referred as ammonia-coated montmorillonite throughout the manuscript. The thermal processing of the ammonia-coated montmorillonite samples was simulated by gradually heating the samples at a rate of 1 K min^−1^ to 320 K (Temperature-Programmed Desorption, TPD). During the TPD phase, the molecules subliming from the surface of the mineral sample were monitored using a Fourier Transform Infrared (FTIR) spectrometer and a reflectron time-of-flight mass spectrometer (ReTOF-MS) coupled with vacuum ultraviolet soft-photoionization (PI) at 10.49 eV. Note that our PI-ReTOF-MS setup only detects selectively ionized molecules subliming directly from the sample’s surface^15,17^. To explore the stability of ammoniated montmorillonite, separate experiments simulated the space weathering of ammoniated montmorillonite over 500 Million years (Supplementary Table 1).

### Infrared spectroscopy

**Fig. 1:**
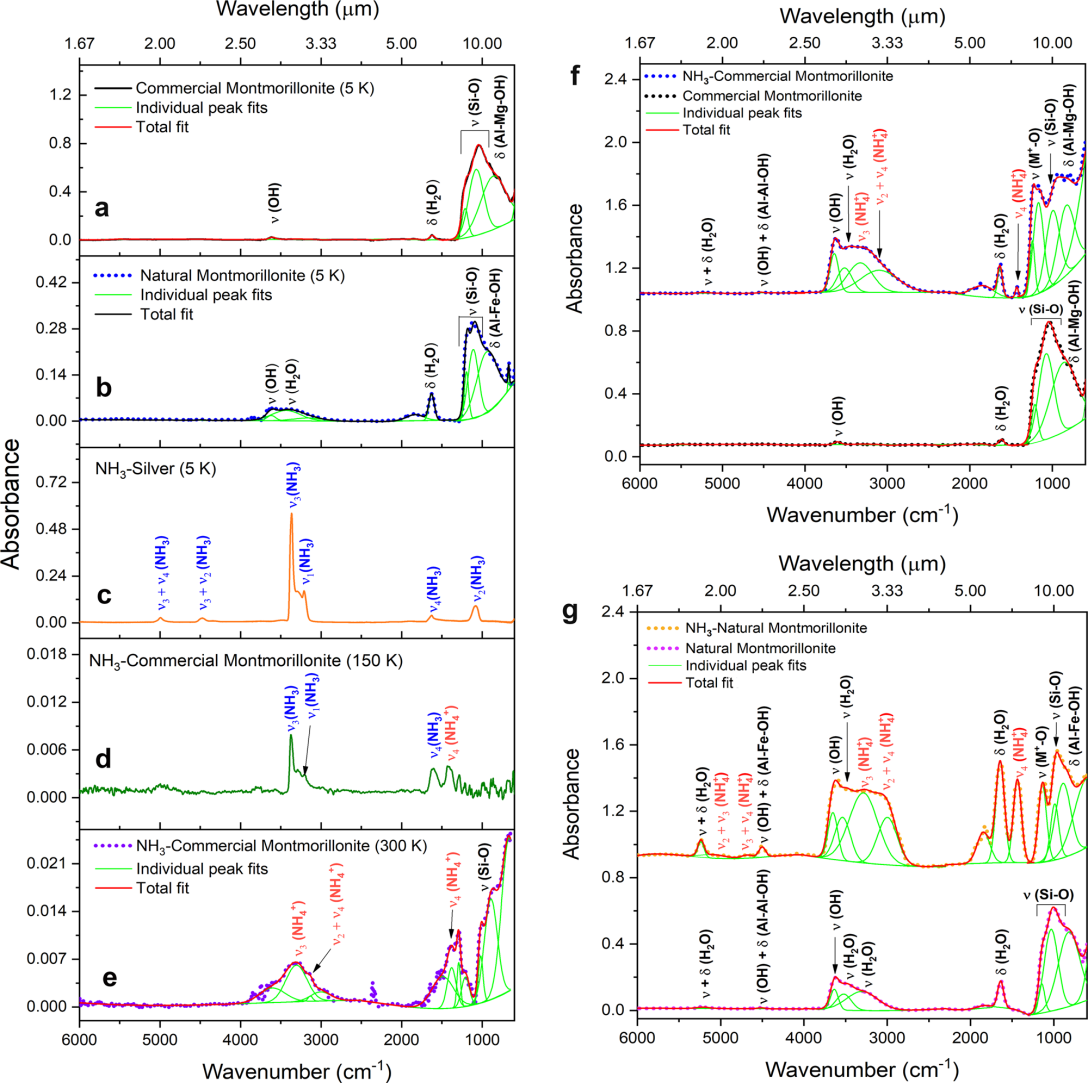
Infrared (IR) spectra of treated and untreated montmorillonite samples with ammonia. In-situ infrared (IR) spectra of (**a**) commercial montmorillonite, (**b**) natural montmorillonite, and (**c**) ammonia (NH_3_) ice on a silver substrate at 5 K. IR spectra of ammonia deposited on the surface of commercial montmorillonite at (**d**) 150 K and (**e**) 300 K. (**f**) and (**g**) are ex-situ IR spectra of ammonia-coated commercial and natural montmorillonite measured at 298 K and 1 atm pressure. Broad IR spectra are deconvoluted to identify the individual peak positions. Vibrational modes of chemical species are labeled using symbols ‘ν’ and ‘δ’. Detail assignments of the bands are provided in Supplementary Tables S2–S5. In spectra (**d**) and (**e**) the initial mineral absorptions are subtracted to clearly identify absorptions of ammonia and ammonium ions.

**Fig. 2:**
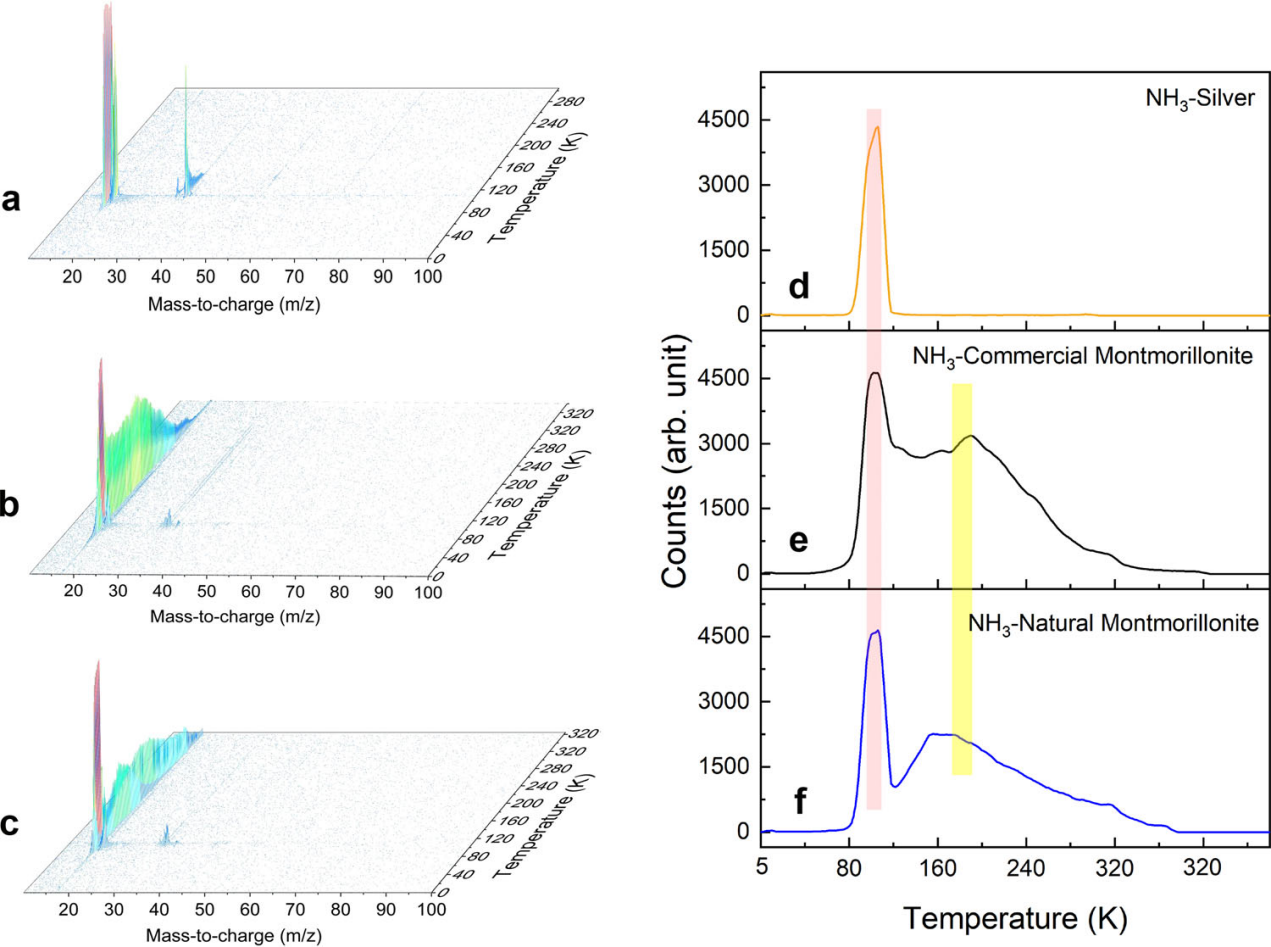
PI-ReTOF mass spectra measured during the TPD (temperature programmed desorption) phase at a photoionization energy of 10.49 eV. 3D mass spectra of subliming molecules from the surface of (**a**) silver substrate, (**b**) commercial and (**c**) natural montmorillonite. TPD profiles of ammonia measured at mass-to-charge ratio (m/z) of 17 in the mass spectra of ammonia coated (**d**) silver, (**e**) commercial montmorillonite and (**f**) natural montmorillonite. The pink and yellow color bars indicate first and second sublimation events respectively. Temperature increase was halted at 320 K till all the molecules sublimed.

The FTIR spectra of commercial (Fig. 1a) and natural (Fig. 1b) montmorillonite reveal broad vibrational features corresponding to Si-O stretching and Al-Mg-OH bending modes near 1000 and 840 cm^−1^, respectively. The Si-O and Al-O linkages contribute to the structural integrity of montmorillonite by forming two dimensional, linked tetrahedral and octahedral sheets. The OH groups bonded with aluminum ions show absorptions near 3621 cm^−1^. The FTIR spectrum of pure ammonia ice at 5 K (Fig. 1c) shows characteristic fundamentals at 3369 (ν_3_; N-H asymmetric stretch), 3209 (ν_1_; N-H symmetric stretch), 1625 (ν_4_; N-H bending), and 1091 (ν_2_; N-H wagging) cm^−1^ along with combination bands and overtones at 4992 (ν_3_ + ν_4_), 4476 (ν_3_ + ν_2_) and 3290 (2ν_4_) cm^−1^ (Supplementary Table 2)^18^. TPD studies on pure ammonia (NH_3_) reveal that its sublimation profile starts at 80 K and peaks at around 100 K (Fig. 2a and d); these temperatures agree well with previous studies of pure ammonia systems^19^. The absence of any absorption features of ammonia in the FTIR spectra recorded at 150 K is well explained by the aforementioned sublimation profile.

Quite remarkably, when ammonia-coated commercial montmorillonite sample is warmed up, vibrational features of ammonia are still detectable even at 150 K (Fig. 1d) implying that ammonia molecules are still adsorbed on the surface of the mineral. At this temperature, a new band appears at 1420 cm^−1^, which could be linked to the bending mode (ν_4_) of the ammonium ion (Fig. 1d)^8,20^. A further increase of the temperature to 300 K reveals new broad absorption features in the region of 3500–2850 cm^−1^ (Fig. 1e) and a decrease in the intensity of ammonia bands. Peak fitting suggests that the bands centered at 3272 and 3100 cm^−1^ are attributed to the asymmetric stretching vibration (ν_3_) and combination bands (ν_2_ + ν_4_), respectively, of NH_4_^+^ ions^8,14,20^. The bending mode (ν_4_) of ammonium ions at around 1420 cm^−1^ is also enhanced at 300 K. The broad feature in the 1297–1000 cm^−1^ region could be attributed to perturbations in the crystal lattice of phyllosilicate. We performed an identical experiment with the ammonia-coated natural montmorillonite sample. The FTIR spectra recorded during the warm-up phase revealed a decrease in the intensity of ammonia bands (Supplementary Fig. 3) and the appearance of new vibrational features corresponding to NH_4_^+^ ions (Supplementary Fig. 4); which are in very well agreement with that observed for ammonia-coated commercial montmorillonite sample.

**Fig. 3:**
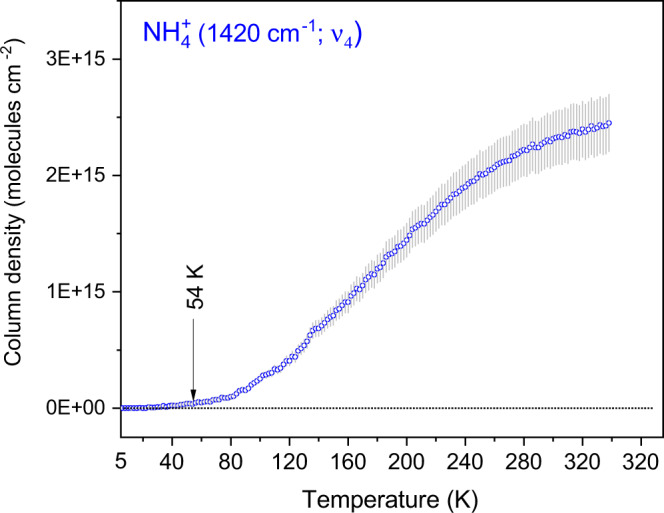
Evolution of column density of NH_4_^+^ ion. Column density at 7.04 µm (1420 cm^−1^; ν_4_) band was measured as a function of temperature during the TPD (temperature programmed desorption) phase of ammonia-coated montmorillonite. Column density was calculated using the calculated absorption co-efficient (1.50 × 10^−16^ cm molecule^−1^) of bending mode (ν_4_) of NH_4_^+^ ion reported in the reference^49^. The vertical error bars indicate standard deviation (±10%).

Beyond the in-situ measurements (i.e., inside the experimental chamber and in UHV conditions), we also collected FTIR spectra of the ammonia-coated montmorillonite samples (commercial and natural) ex-situ (i.e., outside of the UHV chamber, at 298 K at 1 atm) and compared their spectroscopic data with that of pure (untreated) commercial and natural montmorillonites (Fig. 1f and g). The commercial montmorillonite sample treated with ammonia shows N-H stretching (ν_3_), combination (ν_2_ + ν_4_) and bending (ν_4_) modes of NH_4_^+^ at 3270, 3090 and 1430 cm^−1^ respectively. These assignments are in excellent agreement with reported data of the vibrations of ammonium ions bonded with montmorillonite (Supplementary Tables 3–5)^20,21^. Absorption bands of water (H_2_O) present in the mineral are observed at 5230 (ν + δ), 3458 (ν) and 1637 cm^−1^ (δ); this is consistent with the crystallographic studies of montmorillonite suggesting a water presence in the interlayers of the Si-O and Al-O lattice^22^. Bands at 3621 and 1847 cm^−1^ correspond to metal cation-OH vibrations [ν(OH)]^21^. Once again, these findings correlate well with the ex-situ FTIR data of ammonia-coated natural montmorillonite sample (Fig. 1g) displaying the (ν_3_ + ν_4_), (ν_2_ + ν_3_), ν_3_, (ν_2_ + ν_4_), and ν_4_ vibrational modes of ammonium ions at 4705, 4969, 3263, 3070, and 1430 cm^−1^ respectively. For clarity, magnified images of the spectral regions 4000–2500 and 1700–1300 cm^−1^ from Fig. 1f and g are shown in the supplementary Figs. 5 and 6. Intriguingly, the spectrum of natural montmorillonite treated with ammonia in our experiment resembles remarkably well with the reference ammoniated montmorillonite sample synthesized by Ferrari et al.^8^ at room temperature and pressure (Supplementary Fig. 7). Based on these results, we conclude that FTIR spectroscopy provides compelling evidence on the formation of ammoniated-phyllosilicates via thermal processing of ammonia ice deposited on the surface of the mineral$.$ To collect quantitative information on the mechanism, we recorded FTIR spectra during the TPD phase of the ammonia-coated montmorillonite and observed the evolution of ν_4_ bending mode absorption of NH_4_^+^ with increasing temperature (Supplementary Fig. 8). We integrated the ν_4_ mode absorption of NH_4_^+^ to determine the column density (N; molecules cm^−2^) using a modified Lambert–Beer equation (Supplementary Note 2)^23^. The column density plot as a function of temperature (Fig. 3) reveals that the ammonium ion forms within the signal-to-noise of our experiment at 54 ± 6 K under our experimental conditions. An active acid-base chemistry involving proton exchange could occur at very low temperatures. For instance, Jamieson et al. reported the deprotonation of hydrogen cyanide (HCN) in ices at temperatures as low as 23 K^24^. Thus, the formation of ammonium ions on the mineral’s surface could be triggered at low temperature by proton transfer from the mineral to adsorbed ammonia molecules.

Infrared spectroscopy also revealed a significant difference in the amount of interlayer water present in the natural and commercial montmorillonite samples. The column density (N) of interlayer water determined at the 1637 cm^−1^ band [δ(H_2_O)] for non-ammoniated commercial and natural montmorillonites revealed that the latter has a higher concentration N = 2.3 ± 0.2 × 10^17^ molecules cm^−2^) of interlayer water molecules in contrast to the former (N = 1.1 ± 0.1 × 10^17^ molecules cm^-2^, Supplementary Fig. 9). It is important to note here that the column density of bending mode (ν_4_) of NH_4_^+^ ion is also higher for ammonia-coated natural montmorillonite (N = 2.2 ± 0.2 × 10^15^ molecules cm^-2^) compared to ammonia-coated commercial montmorillonite (N = 1.5 ± 0.1 × 10^15^ molecules cm^−2^). Thus, it could be argued that the amount of ammonium ions formed is proportional to the water content of the mineral. Interlayer water molecules in montmorillonite have been observed to be a potential hydrogen donor due to their more dissociative nature compared to free water^25^. These observations suggest that higher water content of natural montmorillonite enhances the extent of ammonium ion formation by increasing the proton availability.

To measure the efficiency of this acid-base reaction we determined the column density of 3369 cm^−1^ band of ammonia at 5 K (N = 6.3 ± 0.6 × 10^16^ molecules cm^−2^) and 300 K (N = 1.4 ± 0.2 × 10^16^ molecules cm^−2^) in ammonia-coated natural montmorillonite sample (Supplementary Fig. 3). From this data, we can imply that 78% of ammonia is absent from the surface, some molecules desorb from the surface and some are converted into NH_4_^+^ ions during the TPD phase. The column density of ν_4_ mode of ammonium ion in ammonia-coated natural montmorillonite sample is only 2.2 ± 0.2 × 10^15^ molecules cm^−2^. This suggests that out of total ammonia absent from the surface (4.9 ± 0.5 × 10^16^ molecules cm^−2^) only 4.5% is converted into ammonium ions, the rest 95.5% desorbs from the surface during the TPD phase.

### Mass spectrometry

The findings from FTIR spectroscopy are also supported by PI reflectron time-of-flight mass spectrometry (PI-ReTOF-MS). Figure 2a–c depict the temperature dependent mass spectra (10–100 amu range) of subliming molecules of the control experiment where ammonia was deposited directly on the silver substrate, along with data from the experiments in which ammonia was deposited on the commercial and natural montmorillonite samples, respectively. The corresponding TPD profiles of ammonia collected at mass-to-charge ratio (m/z) of 17 are depicted in Fig. 2d–f. The TPD profile of ammonia (NH_3_) desorbing from the surface of silver reveals a single sublimation event ranging from 80 K to 120 K, centered at 100 K, while the TPD profiles of ammonia desorbing from the surface of commercial and natural montmorillonite revealed bimodal graphs with two sublimation events (Fig. 2e and f). The first sublimation events start at 80 K and are centered at 100 K; this resembles the sublimation profile of neat ammonia ice and therefore can be attributed to the sublimation of ammonia ices. The broad, second sublimation events are spread over the temperature range from 120 to 320 K covering the surface temperatures of Ceres of 180 to 240 K. These broad features are attributed to desorbing ammonia molecules, which were adsorbed at the surface of montmorillonite samples through non-covalent interactions with the structural hydroxy groups (HO-Al) or oxygen atoms of the silicate framework^26,27^.

## Discussion

**Fig. 4:**
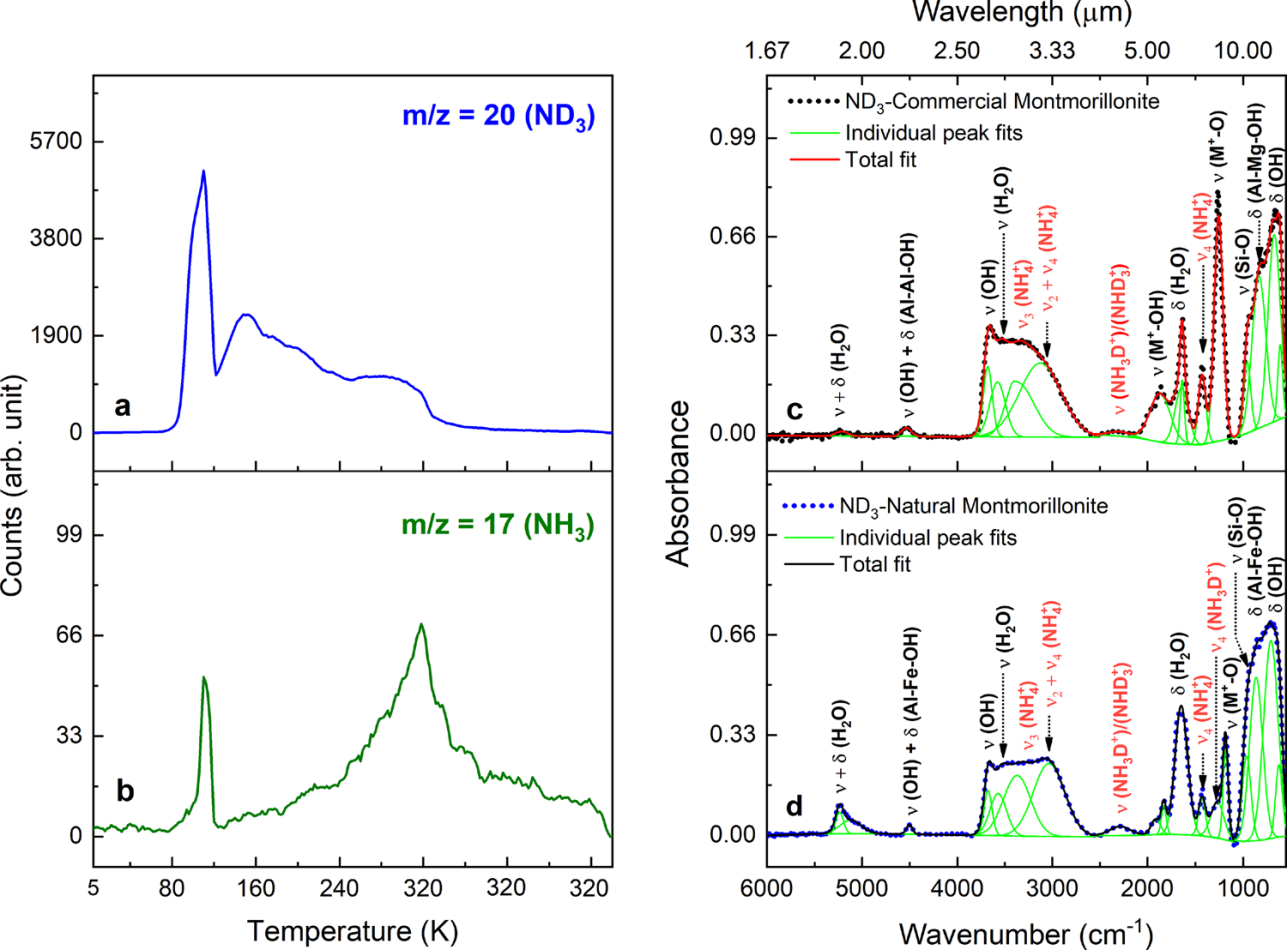
PI-ReTOF mass and infrared (IR) spectra of D3-ammonia (ND_3_) treated montmorillonite samples. TPD (temperature programmed desorption) profiles measured at mass-to-charge ratio (m/z) of (**a**) 20 and (**b**) 17 in the mass spectrum of ND_3_ coated natural montmorillonite at a photoionization energy of 10.49 eV. D3-ammonia (ND_3_) and ammonia (NH_3_) are assigned to the ion signals at m/z = 20 and m/z = 17 respectively. Deconvoluted infrared spectra of D3-ammonia treated (**c**) commercial and (**d**) natural montmorillonite. Vibrational modes of chemical species are labeled using symbols ‘ν’ and ‘δ’.

Having unraveled the existence of ammoniated-phyllosilicates via thermal processing of low temperature ammonia ice on surfaces of phyllosilicates, we are now attempting to unravel the underlying formation mechanisms. Experiments with naturally occurring montmorillonite were repeated with fully deuterated ammonia (D3-ammonia, ND_3_). The PI-ReTOF-MS data of these systems revealed signals at m/z = 20 as well as at m/z = 17 (Fig. 4a, b). It is critical to recall that at a photon energy of 10.49 eV, gas phase ammonia and D3-ammonia do not fragment^28^. Therefore, the signal at m/z = 20 and m/z = 17 must be linked to ND_3_ and NH_3_ molecules, respectively. Considering that only D3-ammonia was deposited onto montmorillonite, the thermal processing during the TPD phase likely resulted in a deuteron vs. proton exchange. These protons are most likely to originate from the interlayer water of the montmorillonite. Note that the m/z = 17 ion counts at 320 K suggests an enhanced activation energy to successively exchange three deuterons by three protons. It is important to mention that in a control experiment, in which D3-ammonia was admitted into the UHV system, no signal was detected at m/z = 17. Further, an ex-situ FTIR analysis of the samples revealed a new absorption in the region 4.0–4.65 µm (2500–2150 cm^−1^; Fig. 4c, d), which is absent in the ammonia treated montmorillonite. This broad feature can be associated with the stretching modes of partially deuterated ammonium ions (NH_3_D^+^ or NHD_3_^+^). These data are in good agreement with the N–D stretching frequencies of deuterated ammonium salts^29^. Therefore, we can conclude that FTIR and PI-ReTOF-MS analysis provide solid evidence on a thermally activated proton exchange between interlayer water of the montmorillonite and (D3) ammonia. We have also performed experiments at a deposition temperature of 40 K, which is more realistic to the Solar System temperature. The IR spectrum of ammonia ice at 40 K and 5 K are alike (Supplementary Fig. 10). Similarly, the desorption profile of ammonia (Supplementary Fig. 11) and the evolution of column density of ammonium ions (Supplementary Fig. 12) measured as a function of temperature during the TPD phase, are in agreement with that of recorded after deposition at 5 K. The ex-situ IR spectra of the ammonia-coated montmorillonite samples prepared after condensation at 5 K and 40 K revealed similar vibrational features of ammonium ions (Supplementary Fig. 13). These results suggest that the deposition of ammonia in the range of 5 K to 40 K on montmorillonite does not affect the ammoniation process.

Finally, we also explored the effects of space weathering on the ammoniated montmorillonite at 5 K and 150 K (Supplementary Fig. 14). This was achieved by irradiating the samples with energetic electrons, which mimic the secondary electrons generated in the track of galactic cosmic rays and solar wind protons^30,31^ interacting with Ceres’ surface over 500 Million years. Although Ceres formed along with other solar system bodies, certain regions of the Ceres’ surface could be relatively new, only a few million years old. For instance, a recent study suggests that Ceres’ cryovolcano Ahuna Mons and its craters evolved within the past 210 ± 30 million years^32^. Similarly, it is estimated that the Occator crater region of Ceres is 1.6–63.7 million years old^33^. However, the fact that the majority of Ceres’ surface is older than the fresh crater regions cannot be neglected^34^. Nevertheless, the choice of 500 million years to investigate the effect of the cosmic rays on the stability of ammoniated minerals is still reasonable. Surprisingly, the FTIR spectra of irradiated and non-irradiated ammoniated montmorillonite are indistinguishable. These findings suggest that space weathering—at least over 500 Million years—has a minimal effect on the stability of ammoniated-phyllosilicates.

In conclusion, our FTIR and PI-ReTOF-MS studies deliver conclusive evidence that ammoniated-phyllosilicates on Ceres surface can be the result of thermally activated proton transfer processes between ammonia and interlayer water molecules within phyllosilicate minerals. In the present experimental approach, the hypothesis of the existence of phyllosilicates minerals prior to the condensation of ammonia ice is driven by recent simulations which suggest that phyllosilicates could have been formed at the dust agglomeration stage in the protoplanetary disk and could have been transferred to planetary bodies^35,36^. Intriguingly, Ceres-like airless bodies could accrete phyllosilicate rich dust grains during their genesis in the solar nebula. However, the location where it accreted dust grains is quite debatable. The widespread observation of ammoniated phyllosilicates of Ceres’ regolith and water-rich ice mantle makes this dwarf planet distinct from alternative main belt bodies such as C-type asteroids. Therefore, it has been theoretically proposed that Ceres may not have formed at its current location^37,38^. Ceres might have originated at a greater heliocentric distance where ammonia ices on the surface would have been stable^37^, before being transported to its current location. For instance, evidence of ammonia ice on Saturnian region objects has been documented such as on Enceladus^39^. Alternatively, Ceres could have evolved near to its current location by accreting particles; these pebble-sized particles must have been drifted from large heliocentric distances where ammonia ice was stable^40^. In both scenarios, strong non-covalent interactions such as hydrogen bonding between ammonia molecules and HO-Al/Si-O-Si moieties of the mineral lattice could facilitate the adsorption of ammonia on the mineral’s surface. This is evident from the FTIR and mass spectra of ammonia-coated montmorillonite samples, which display vibrational features corresponding to ammonia and mass signal at m/z = 17 respectively, over the broad temperature range (150–320 K). A recent computational study investigating the adsorption mechanism of ammonia on the surface of kaolinite—a phyllosilicate mineral, support the evidence of surface adsorption of ammonia through hydrogen bonding with the structural hydroxy groups (H_2_N-H….OH-Al) of the mineral^41^. The adsorb ammonia molecules could undergo hydrogen exchange with the water molecules present in the interlayers of the Si-O and Al-O mineral framework. Previous investigations suggest that interlayer water molecules of montmorillonite are more acidic in nature and therefore, potential hydrogen donors compared to Al-OH groups^25,42^. Comparison of IR spectra of non-ammoniated commercial and natural montmorillonite samples revealed that the latter has a higher concentration of water molecules in the interlayers of the mineral lattice. This difference in water content is also reflected in the extent of ammoniation of the two samples. IR spectra of the NH_4_^+^-natural montmorillonite display a higher column density of bending absorption band of NH_4_^+^ relative to the commercial montmorillonite. The higher water content of natural montmorillonite enhances the extent of ammonium ion formation by increasing the proton availability. With ammonia being more basic relative to interlayer water, this acid-base reaction proceeds easily even at low temperatures as demonstrated in the present work. The ammonium ions formed through acid-base base reaction can then replace the exchangeable metal cations (K^+^ and Na^+^) of the mineral. Overall, these experiments suggest that the reaction between ammonia and phyllosilicates represents a feasible and highly probable mechanism to form ammoniated-phyllosilicates on Ceres^4^ as observed by the Dawn spacecraft^43^ and ground-based telescopes such as the NASA IRTF on Mauna Kea^4^. The present study can also be implied to asteroids such as 10 Hygiea and 324 Bamberga, which reveal infrared absorptions near 3 µm region like Ceres that could be a hint of the presence of ammonium bearing minerals^20,44^.

## Methods

### Simulation experiments

The experiments were conducted in an ultra-high vacuum chamber evacuated to a base pressure of a few 10^−10^ torr using magnetically levitated turbo molecular pumps backed by oil-free dry scroll pumps^15,16,45^. Powdered samples of commercially available montmorillonite (Sigma-Aldrich; grain size <20 µm) and naturally occurring montmorillonite (Source Clays Repository of the Clay Minerals Society (CMS); grain size <36 µm) were pressed separately onto a silver substrate to form a uniform layer of 50 ± 5 µm and 80 ± 10 µm for commercial and natural montmorillonite, respectively. The grain size of Ceres’ average regolith determined from the VIR data is ~100 µm^46^. The grain size of montmorillonite samples used in this study is smaller (20–36 µm) than that of the average regolith of Ceres. Although smaller grain size particles could adsorb more ammonia molecules due to a higher volume-to-surface ratio compared to coarser particles, the mechanism behind the ammoniation process will not change due to the difference in the grain size. Larger grain size particles could exhibit higher absorbance in certain regions of the spectrum as light can penetrate deeper in the sample^47^. The mineral samples were mounted separately on a cold finger and kept inside an UHV chamber for ~48 h at room temperature so any volatiles would sublime. Then, the cold finger was cooled to 5.0 ± 0.2 K using a closed cycle helium refrigerator (Sumitomo Heavy Industries, RDK-415E). Ammonia gas was deposited through a glass capillary array onto the cold samples (either silver substrate, natural, or commercial montmorillonite) to develop ammonia-ice on the surface of the samples. Note that ammonia was deposited well below its sublimation temperature of 80 K to ensure that majority of the gas molecules would stick into the samples, resulting in the formation of ammonia ice on the samples’ surface instead of contaminating other regions of the UHV chamber. During deposit, the pressure in the main chamber was kept constant at 2 × 10^−8^ torr for a few minutes. The thickness of the ice was determined to be 600 ± 30 nm based on a calibration experiment performed by depositing ammonia gas on a cold silver mirror (see supporting information). The infrared spectra of the ice- NH_3_-covered commercial montmorillonite and NH_3_-covered natural montmorillonite systems were measured in-situ in the mid-infrared (6000–600 cm^−1^) region using a FTIR spectrometer (Nicolet 6700) operated at a resolution of 4 cm^−1^. The obtained IR spectra were fitted to identify the peaks. The baseline of the spectrum was fixed while peaks width and height were free parameters during the fitting. All the peaks were fit using gaussian function. The software runs several iterations till the adjusted R-square value of 1 or close to 1 is achieved. The thermal processing of the ice-mineral system was performed by increasing the temperature of the system to 320 K at a rate of 1 K min^−1^ (TPD); temperature increase is halted at 320 K until most molecules desorbed from the samples and the ReTOF-MS counts are extremely low. During the TPD phase, the desorbing molecules were monitored exploiting a single-PI reflectron time-of-flight mass spectrometer (PI-ReTOF-MS) setup using 10.49 eV ionizing photons. The ions formed due to PI are extracted and eventually separated in the time-of-flight tube based on their mass-to-charge (m/z) ratio before reaching a microchannel plate (MCP) detector. The signal from the MCP detector is then amplified and shaped with a 100 MHz discriminator (Ortec 9305). The discriminator sends the signal to a computer-based multichannel scaler, which records the signal in 4 ns bins triggered at 30 Hz by a pulse delay generator. 3600 sweeps were collected for each mass spectrum per 2 K increase in the temperature during the TPD phase. FTIR spectra were collected during the TPD phase online and in-situ. To simulate the effect of galactic cosmic rays, the ammoniated natural montmorillonite system was exposed to 5 keV energetic electrons at an angle of 70° to the normal of the substrate at an electron current of 100 ± 5 nA for 1 h. Using Monte Carlo simulations computed using CASINO 2.42 software^48^, we estimated that the average penetration depth of the electrons in the ice was 329 ± 80 nm, and the average energy deposited was 10.96 ± 0.10 eV per molecule. Hereafter, ices were annealed at a rate of 1 K min^−1^, and molecules subliming from the substrate were ionized and detected using PI-ReTOF-MS.

### Materials

Commercial montmorillonite (grain size < 20 µm) was procured from Sigma-Aldrich. Naturally occurring montmorillonite was obtained from the Source Clays Repository of the CMS. The untreated natural montmorillonite sample was milled and then sieved using a 36 µm sieve to obtain a fine powder. The referenced ammoniated-natural montmorillonite sample was prepared using the following procedure^8^. The natural montmorillonite mineral was first milled inside a Retsch Centrifugal Ball Mill S 100 employing a 50 ml agate jar, which was spun at 400 rpm for 60 s. The milled sample was sieved using a 36 µm sieve to obtain a very fine powder. The sieved powder sample was kept inside a desiccator for 72 h at 333 K to remove the excess of water. Afterwards, the dried powder of natural montmorillonite was put inside a concentrated ammonia solution (30% NH_3_ in H_2_O) with a mineral/solution ratio of 1:10 wt./volume and shaken for 2 h at 20 Hz. The mixture was left undisturbed for about 150 h. This time was sufficient to allow the cation exchange process to happen and avoid significant changes in the structure/properties of clays due to the high pH of the solution. Later, the mixture was centrifuged to separate the solid fraction from the ammonia solution. The extracted minerals were dried for 24 h at 333 K. Afterwards, the sample was milled, sieved (<36 µm), and stored in a desiccator.

## Supplementary information

Supplementary Information

## Data Availability

The data that support the findings of this study are provided in the main article and its supplementary information files as well as available from the corresponding author upon request. Experimental source data for Figs. 1–4 and Supplementary Figs. 7–13 are provided with this paper.

## Source data

Source Data
