# Supplementary material for: Origin of ammoniated phyllosilicates on dwarf planet Ceres and asteroids
Source: Nat Commun. 2021 May 11;12:2690. doi: 10.1038/s41467-021-23011-4 (PMC8113531; doi:10.1038/s41467-021-23011-4)
Supplement: Supplementary file 1 — Supplementary Information [file 41467_2021_23011_MOESM1_ESM.pdf]

Supplementary Information for

Origin of Ammoniated Phyllosilicates on Dwarf Planet Ceres and Asteroids

Santosh K. Singh[1], *Alexandre Bergantini*[1†], *Cheng Zhu*[1], *Marco Ferrari*[2], *Maria Cristina De Sanctis*[2], *Simone De Angelis*[2], *and Ralf I. Kaiser*[1*]

[1]*Department of Chemistry, University of Hawaii, Honolulu, HI 96822, USA*

W. M. Keck Research Laboratory in Astrochemistrys, University of Hawaii, 96822 HI, USA

[2]*Istituto di Astrofisica e Planetologia Spaziali, INAF, Via del Fosso del Cavaliere 100, Roma, 00133, Italy*

[*]Corresponding author. Email: ralfk@hawaii.edu

[†]Current address: *Federal Center for Technological Education Celso Suckow da Fonseca, Maracanã, 20271110 - Rio de Janeiro, Brazil*

Supplementary Figures

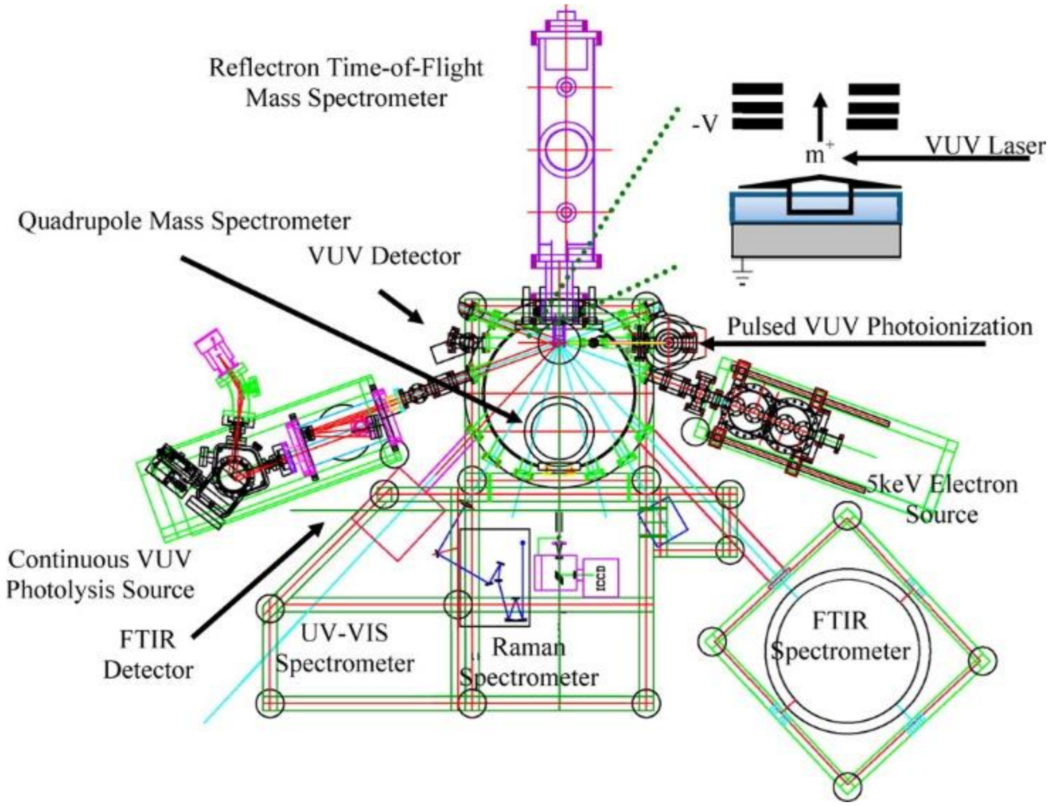

Supplementary Figure 1. Schematic top view of the ultra-high vacuum chamber including the electron source, analytical instruments (FTIR, UV-VIS, ReTOF), and cryogenic target (point of convergence lines)[1-3]. Adapted with permission from ref. 3. Copyright (2015) Royal Society of Chemistry.

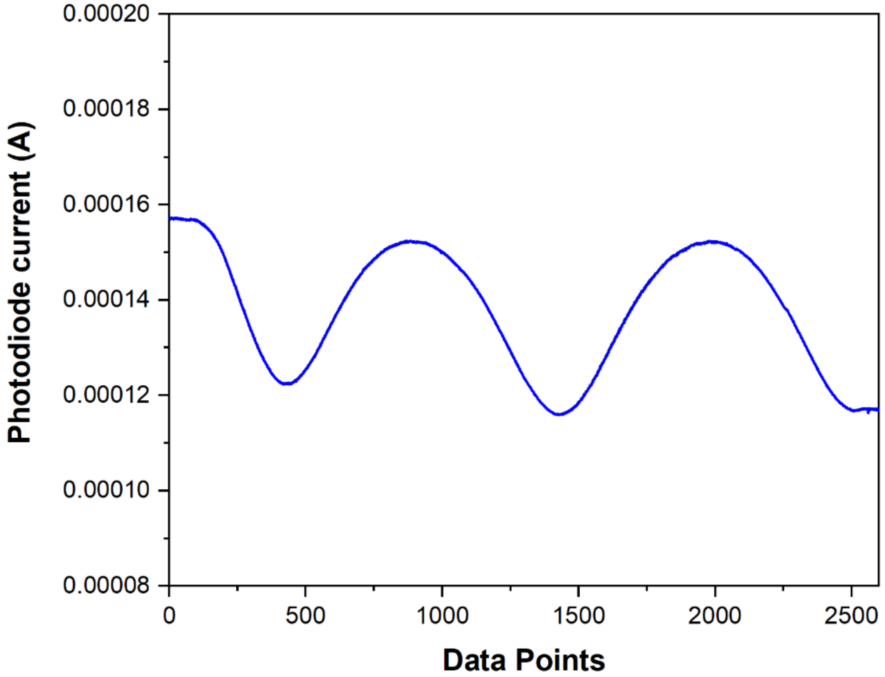

Supplementary Figure 2| Interference pattern measured during the deposition of NH_3 gas for a 632.8 nm laser at an angle of incidence of 4°.

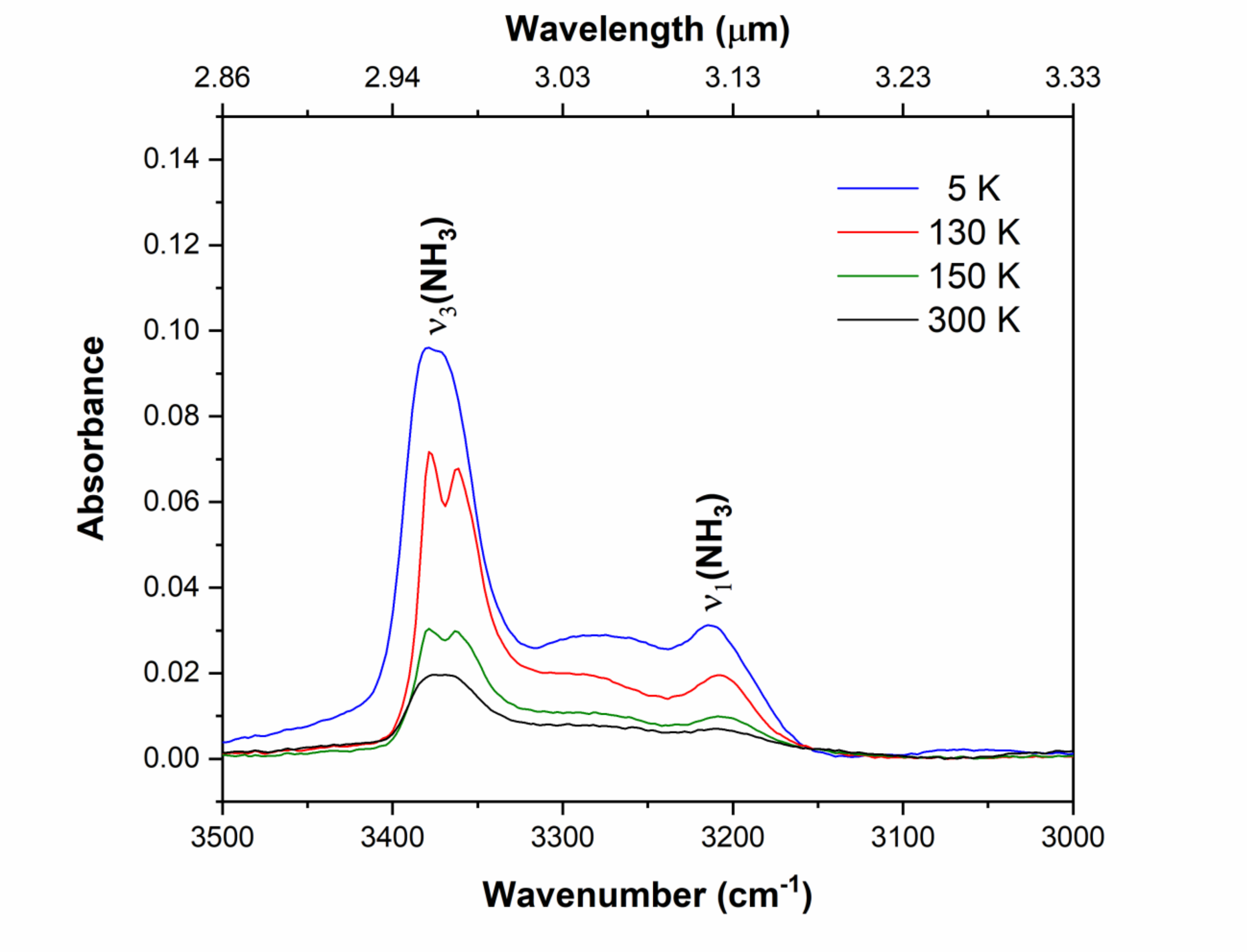

Supplementary Figure 3. Decrease in the absorption of ν_3 (asymmetric stretch) and ν_1 (symmetric stretch) vibrational modes of ammonia (NH_3) ice with increasing temperature recorded during the Temperature-Programmed Desorption (TPD) phase of ammonia-coated montmorillonite. For clarity, the IR spectra measured at only few temperatures are displayed.

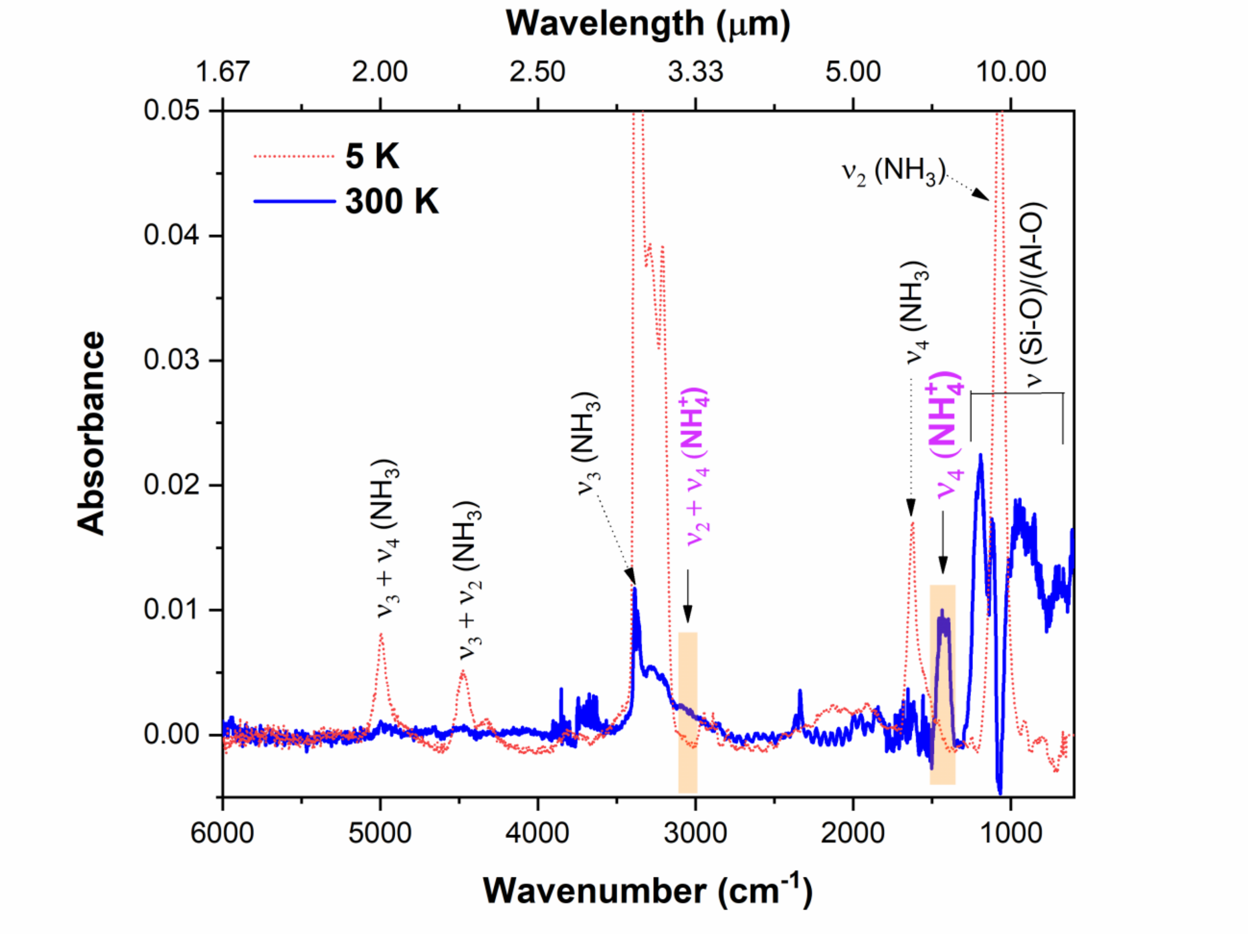

Supplementary Figure 4. In-situ infrared (IR) spectra of ammonia (NH_3) on the surface of natural montmorillonite at 5 K (red) and 300 K (blue). At 300 K new broad absorption features appeared in the region 3150-2800 cm^{-1} and at 1430 cm^{-1} which correspond to ammonium ions (NH_4^+). The initial mineral absorptions are subtracted to clearly identify absorptions of ammonia and ammonium ions. Vibrational modes of ammonia and ammonium ions are labelled using symbol 'ν' in black and pink color fonts respectively. Refer to Supplementary Tables S2 and S4 for detailed assignments of the bands.

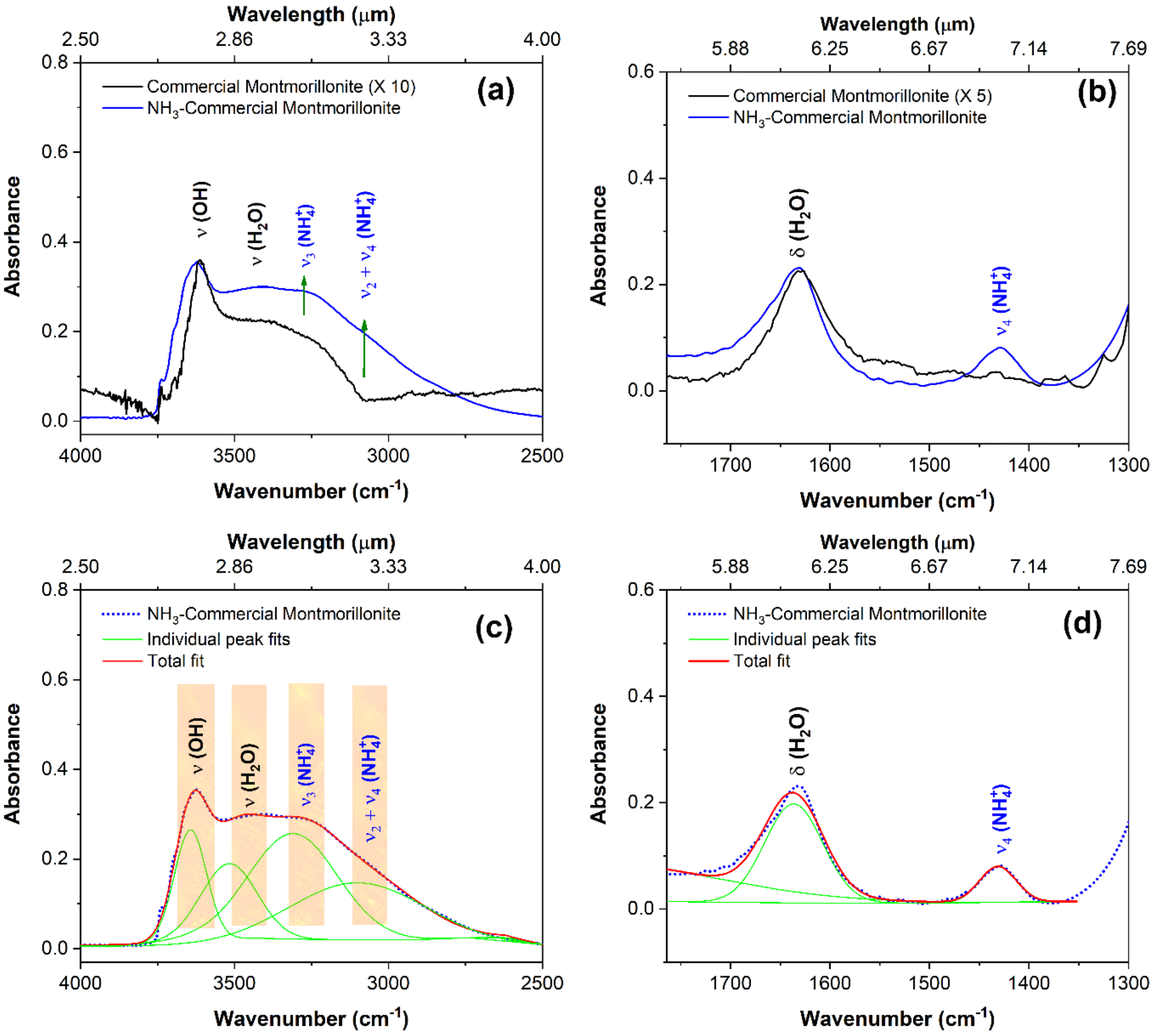

Supplementary Figure 5. Magnified view of the spectral regions (a, c) 4000-2500 and (b, d) 1700-1300 cm^{-1} of the IR spectra displayed in Fig 1f of the main article. Individual peak fits as well as total fit in the IR spectral regions 4000-2500 cm^{-1} and 1700-1300 cm^{-1} of the ammonia treated commercial montmorillonite are displayed in (c) and (d) respectively. Symbols ν_1-ν_4 denote vibrational modes of ammonium ion. Labels ν (H_2O) and ν (OH) indicate stretching vibrational modes of interlayer water molecules and -OH groups bonded to metal ion respectively. Bending vibrational mode of interlayer water molecules are labeled as δ (H_2O). Detail assignments of the bands are provided in Supplementary Tables S3-S5.

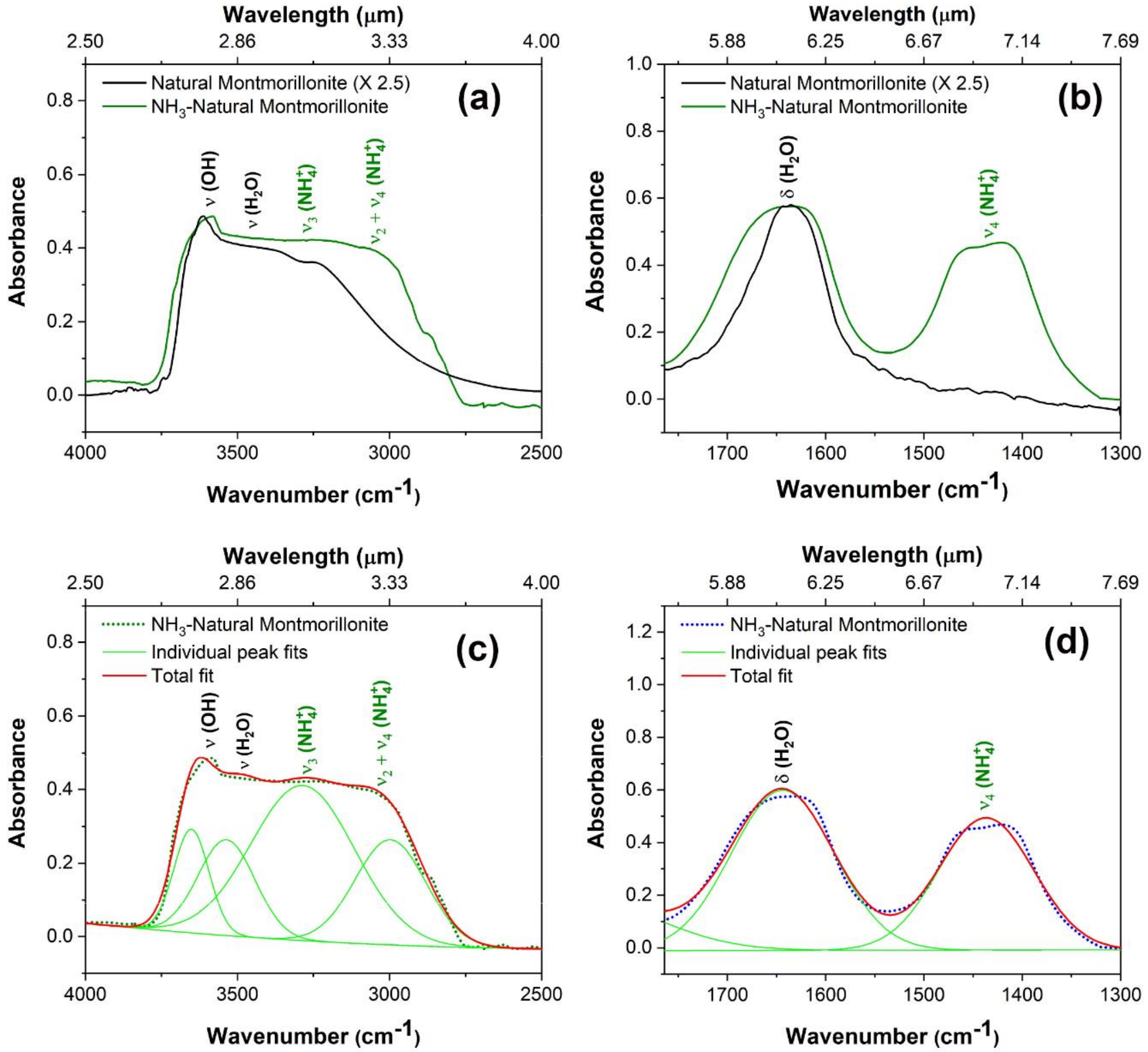

Supplementary Figure 6. Magnified view of the spectral regions (a, c) 4000-2500 and (b, d) 1700-1300 cm^{-1} of the IR spectra displayed in Fig. 1g of the main article. Individual peak fits as well as total fit in the IR spectral regions 4000-2500 cm^{-1} and 1700-1300 cm^{-1} of the ammonia treated natural montmorillonite are displayed in (c) and (d) respectively. Symbols ν_1-ν_4 denote vibrational modes of ammonium ion. Labels ν (H_2O) and ν (OH) indicate stretching vibrational modes of interlayer water molecules and -OH groups bonded to metal ion respectively. Bending vibrational mode of interlayer water molecules are labeled as δ (H_2O). Detail assignments of the bands are provided in Supplementary Tables S3-S5.

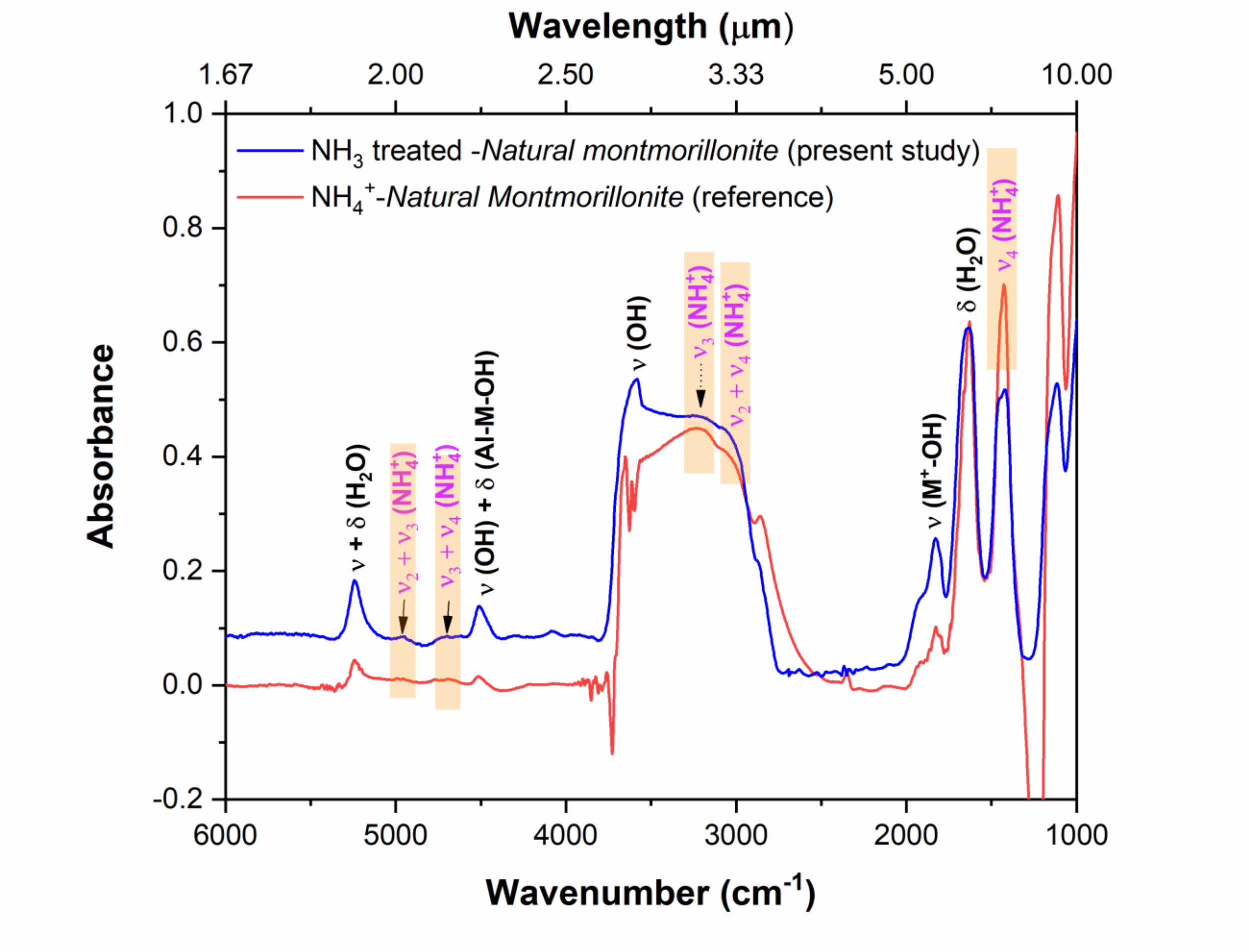

Supplementary Figure 7. Ex-situ infrared spectra of NH_3-coated natural montmorillonite in the present study and reference ammoniated-natural montmorillonite sample prepared following the procedure described in Ferrari et al. *Icarus*, 321, 2019, 522-530.[4] Both the spectra are measured at standard temperature and pressure. Absorption features corresponding to ammonium ions (NH_4^+) are indicated by shaded color bars. See Supplementary Table S4 for detailed assignments.

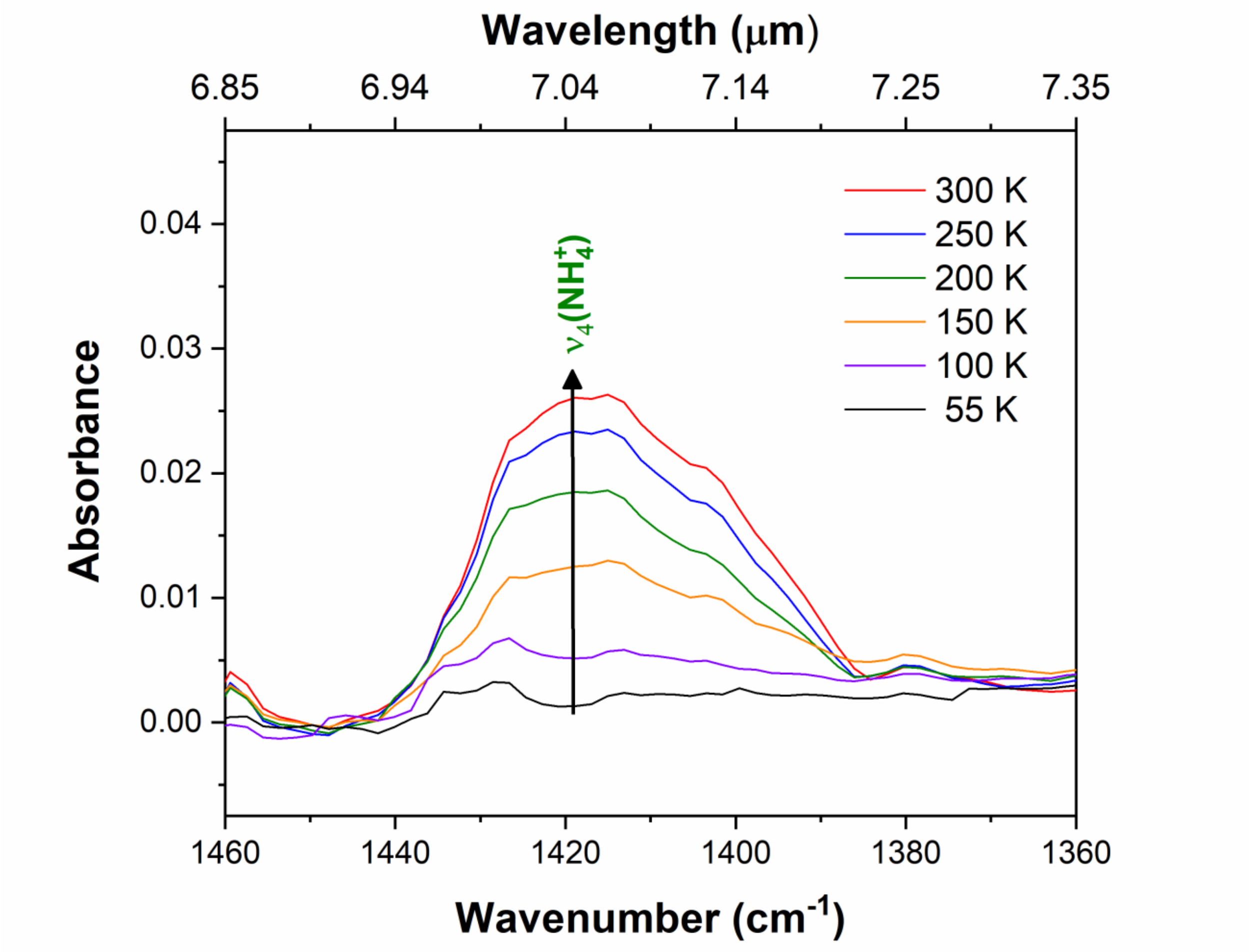

Supplementary Figure 8. Change in the absorption of ν_4 bending mode of NH_4^+ ions with increasing temperature measured during the TPD phase of ammonia-coated natural montmorillonite. For clarity, the IR spectra measured at only few temperatures are displayed.

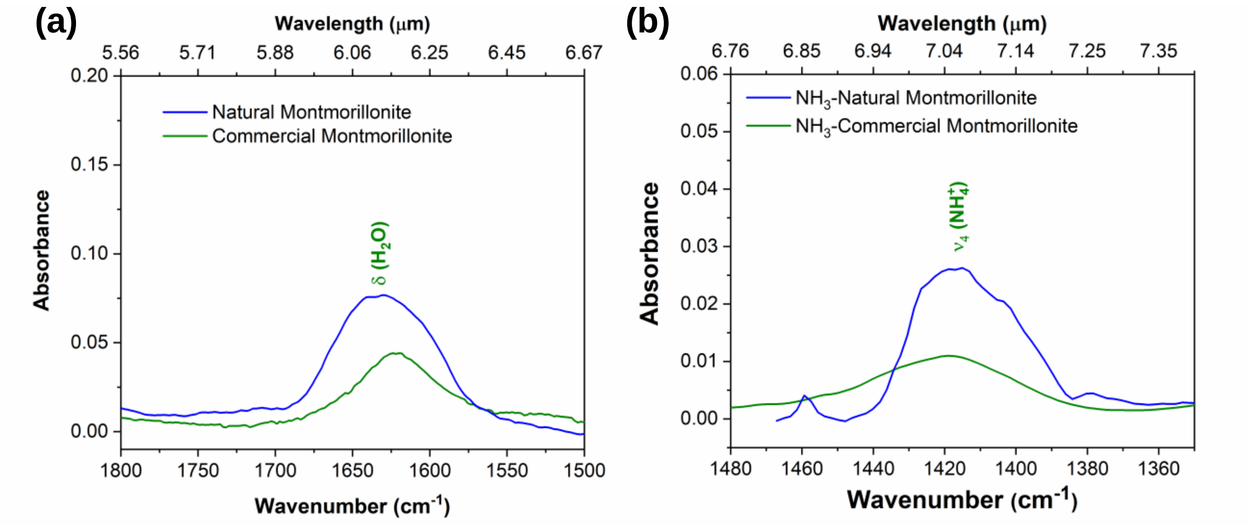

Supplementary Figure 9. (a) Absorption of a bending mode (δ) of interlayer water in non-ammoniated natural and commercial montmorillonite samples. (b) Absorption of ν_4 bending mode of ammonium (NH_4^+) ions in ammonia-coated natural and commercial montmorillonite samples.

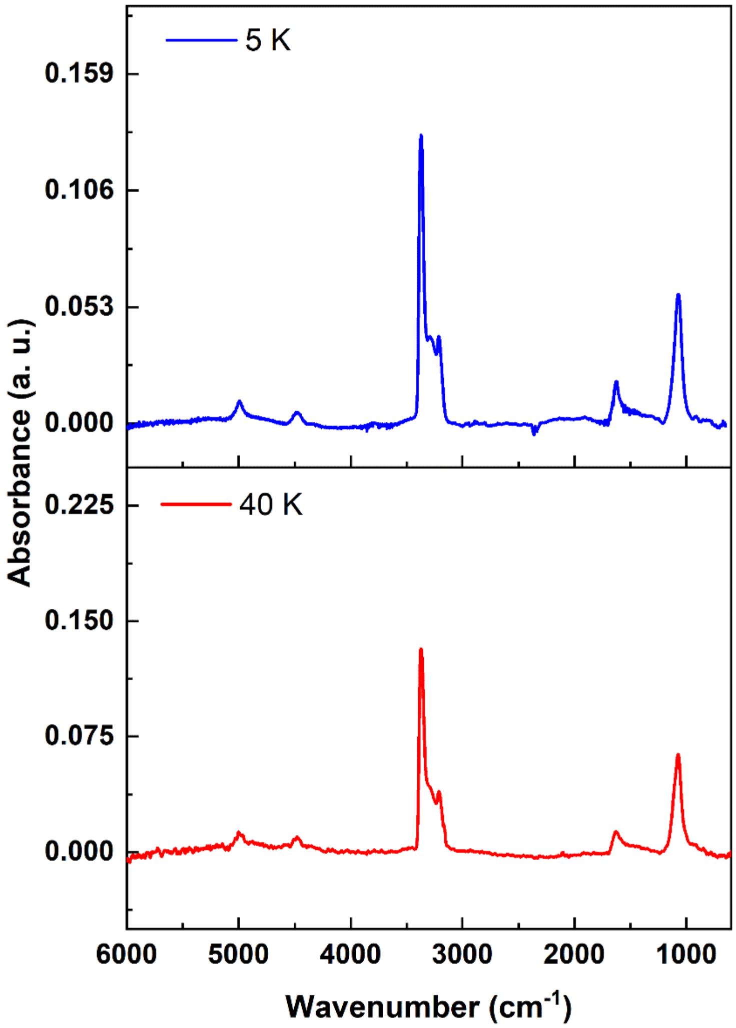

Supplementary Figure 10. IR spectra of ammonia ice on the surface of natural montmorillonite at 5 K and 40 K.

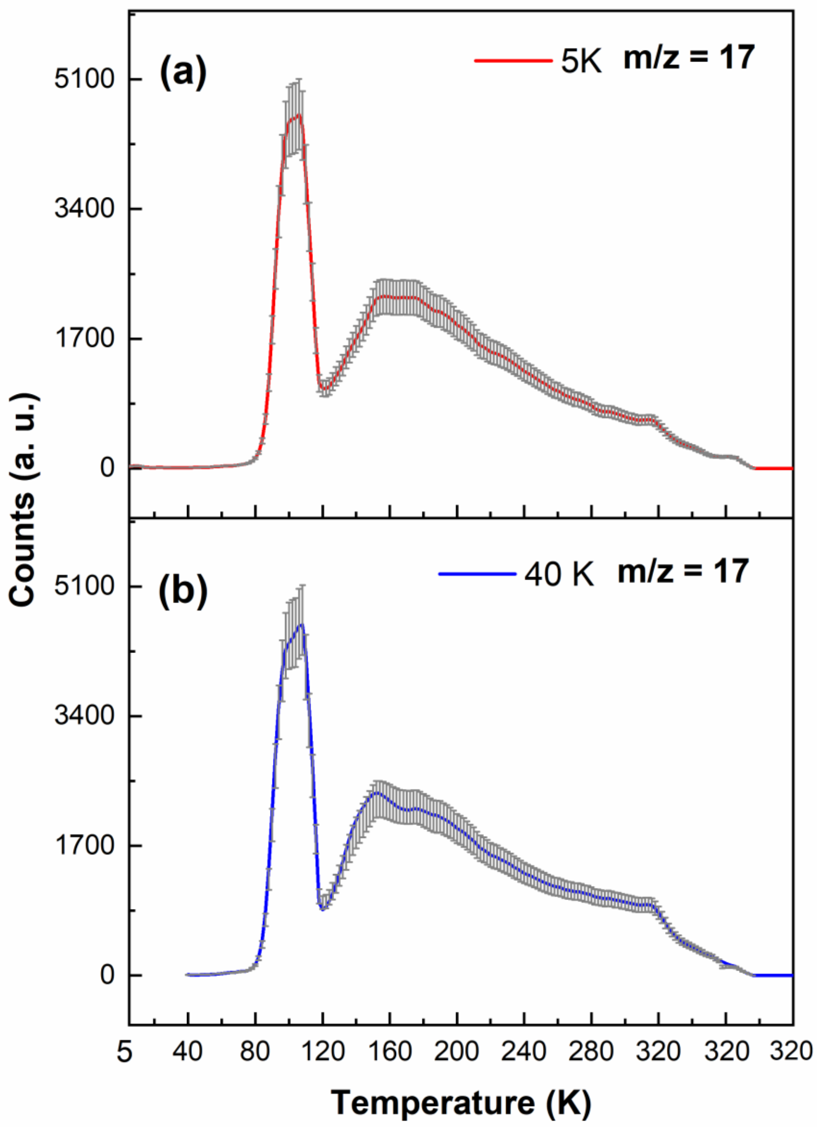

Supplementary Figure 11. PI-ReTOF mass spectra of ammonia (mass-to-charge ratio (m/z) = 17) subliming from the surface of natural montmorillonite during the TPD phase. Measured after deposition of ammonia at (a) 5 K and (b) 40 K at a photoionization energy of 10.49 eV. The vertical error bars indicate standard deviation.

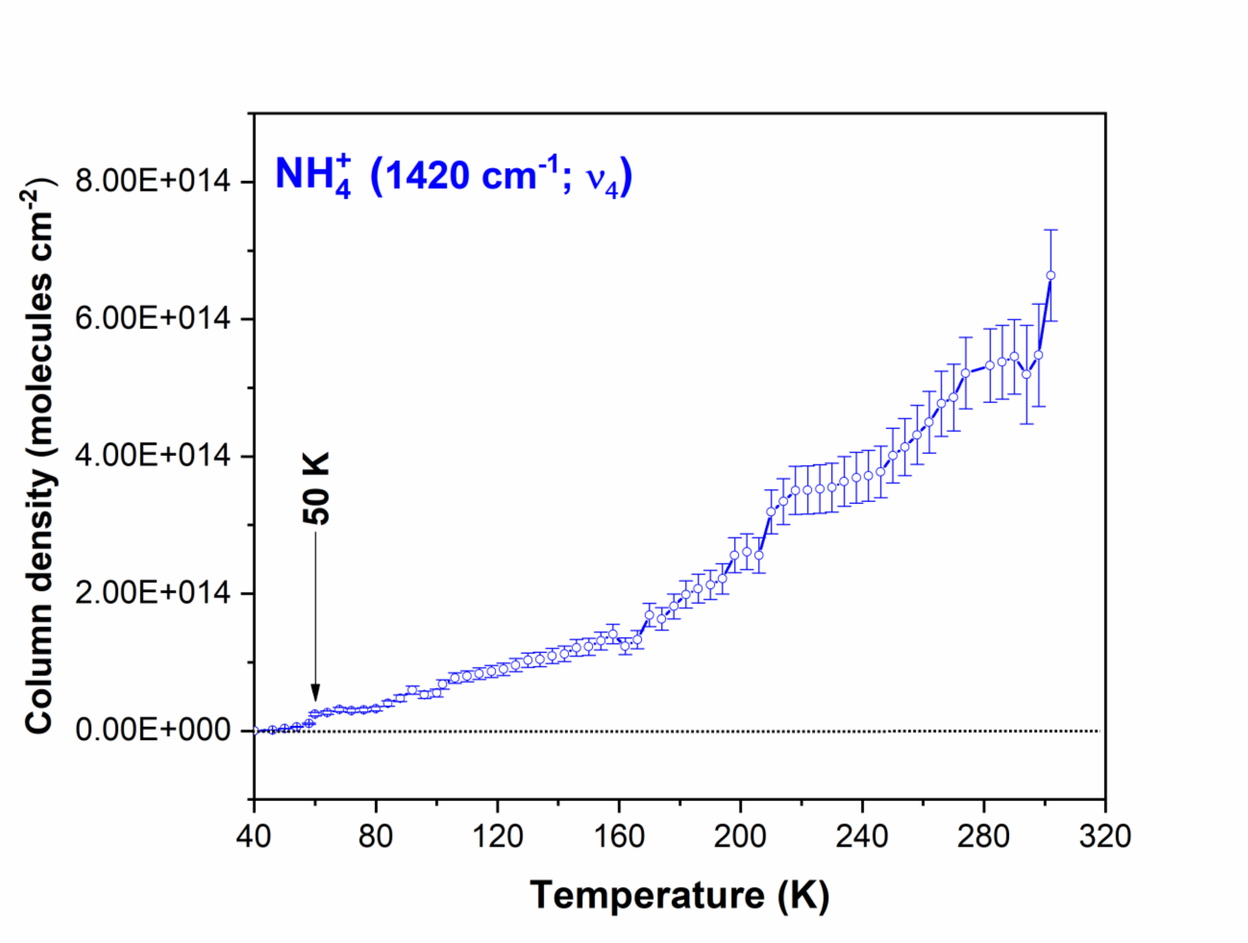

Supplementary Figure 12. Evolution of column density of ammonium ion at 1420 cm^{-1} band measured as a function of temperature during the TPD phase of ammonia-coated natural montmorillonite. Ammonia was deposited at 40 K. The vertical error bars indicate standard deviation (± 10%).

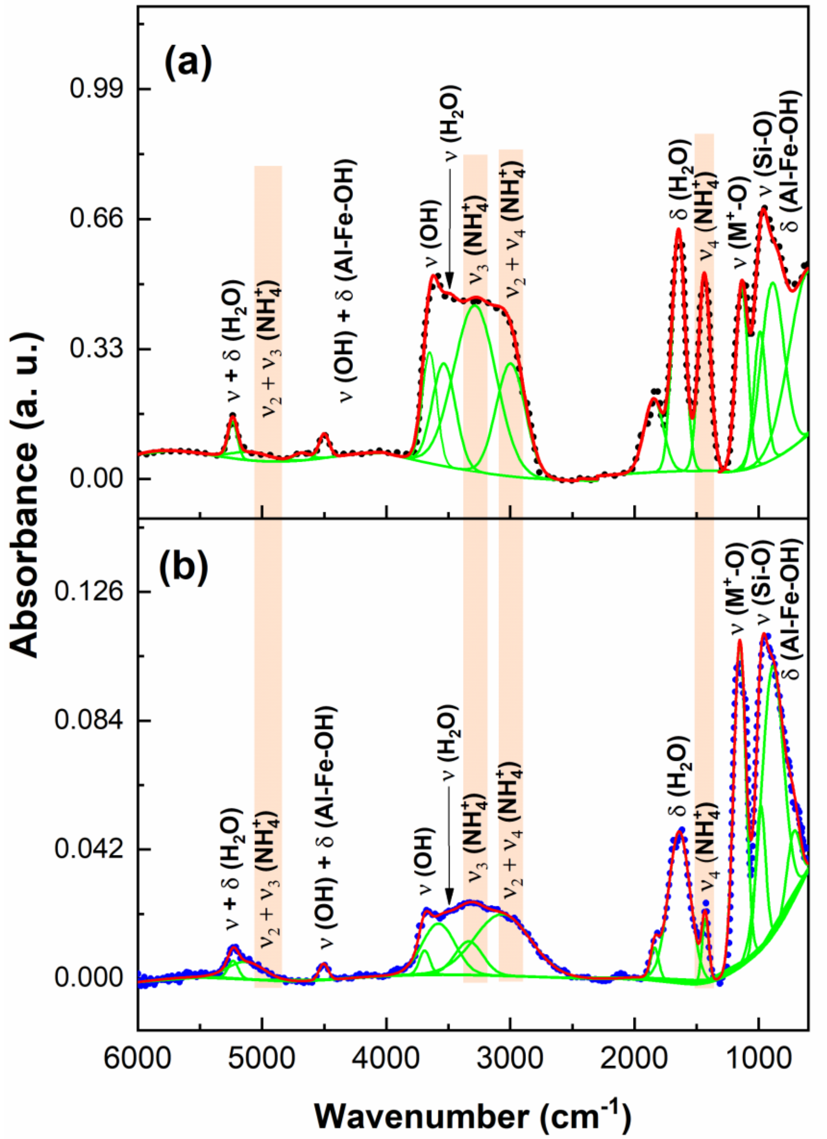

Supplementary Figure 13. Ex-situ IR spectra of ammonia-coated natural montmorillonite samples prepared after deposition of ammonia ice on the surface of natural montmorillonite (a) 5 K and (b) 40 K. Absorptions corresponding to ammonium ions are shaded with color bars. Vibrational modes of chemical species are labeled using symbols ‘ν’ and ‘δ’. Detail assignments of the bands are provided in Supplementary Tables S3-S5.

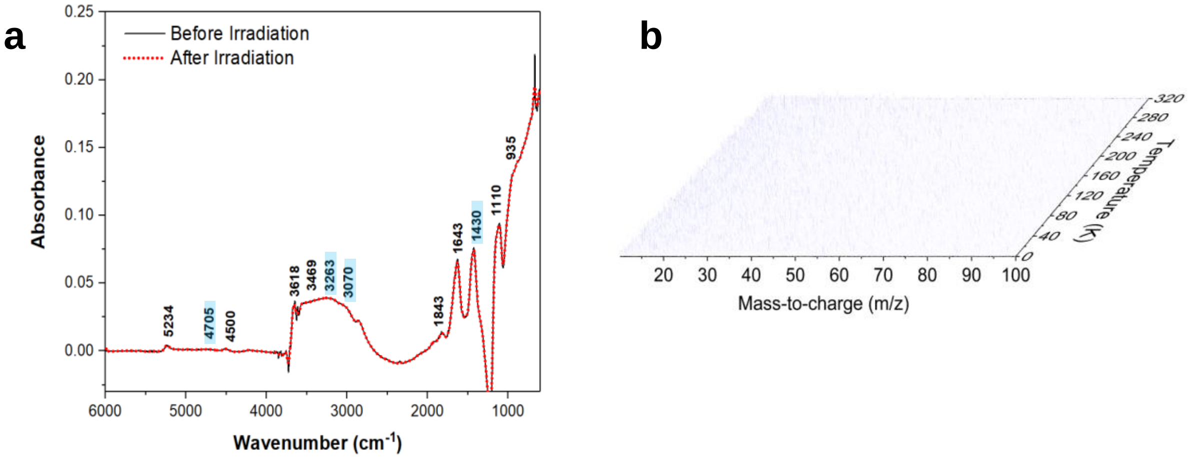

Supplementary Figure 14. (a) Infrared spectra of ammoniated-natural montmorillonite before and after irradiation. Highlighted wavenumbers define the absorptions of NH_4^+ cation. (b) 3D PI-ReTOF mass spectrum measured after irradiation of ammoniated natural montmorillonite at a photoionization energy of 10.49 eV.

Supplementary Tables

Supplementary Table 1. Data applied to calculate irradiation dose per molecule. *Values from CASINO simulations $^{\$}$. Derived values based on 100 nA for 60 min irradiation of NH_3 molecules

Initial Kinetic energy of the electrons	5keV
Irradiation current (I)	100±5 nA
Irradiation time (t)	3600 s
Average penetration depth, l	329.18±80 nm
Average kinetic energy of backscattered electrons, E_{bs}^{*}	3.18±0.3 keV
Fraction of backscattered electrons, f_{bs}^{*}	0.31±0.1
Average kinetic energy of transmitted electrons, E_{trans}^{*},	0.0 keV
Fraction of transmitted electrons, f_{trans}^{*}	0
Density of the ice, ρ	0.74 gcm^{-3}
Irradiated area, A	1 cm^2
total # molecules processed	$(8.63±2)×10^{17}$
dose per molecule, D	10.96 ± 0.1 eV
Total number of electrons generated	$(2.80±0.3)×10^{15}$

Supplementary Table 2. Infrared absorption features of ammonia ice at 5K

Wavenumber (cm^{-1}) observed	Wavenumber (cm^{-1}) literature[5]	Vibrational Assignments	Vibrational Modes
4992	4994	$\nu_3 + \nu_4$	Combination band (asymmetric stretch + degenerated deformation)
4476	4478	$\nu_3 + \nu_2$	Combination band (symmetric stretch + symmetric deformation)
3369	3372	ν_3	Asymmetric N-H stretch
3209	3212	ν_1	Symmetric N-H stretch
1625	1628	ν_4	Degenerated deformation
1070	1097	ν_2	Symmetric deformation

Supplementary Table 3. Infrared absorptions of NH_4^+ ions in ammoniated montmorillonite samples (Natural and Commercial) observed in the present study in comparison with that of reported in the literature.

**Absorption Modes**	**Band position (μm)**					
	This study		Literature			
	Commercial Montmorillonite	Natural Montmorillonite	Berg et al.[6]	Ferrari et al.[4]	Ehlmann et al.[7]	Bishop et al.[8]
$\nu_3 + \nu_2$	-	2.01	2.03	2.01	-	2.03
$\nu_3 + \nu_4$	-	2.12	2.12	2.12	-	2.13
ν_3	3.05	3.06	3.05	3.05	3.04	3.05
$\nu_2 + \nu_4$	3.23	3.25	3.28	3.25	-	3.24
ν_4	6.99	6.99	6.89	6.95	-	6.99

Supplementary Table 4. Infrared absorption features of ammonia treated and untreated natural montmorillonite samples measured at 300 K and 1 atm pressure. Spectra of the samples are provided in Figure 1(g) of the main article.

**NH_3-Natural montmorillonite**			
Wavenumber (cm^{-1})	Wavenumber literature (cm^{-1})[4]	Vibrational Assignments	Carrier
5239	-	ν + δ (H_2O)	Combination band of H_2O
**4969**	**4975**	$\nu_2 + \nu_3$ (NH_4^+)	Combination band (In-Plane bend +Asymmetric stretch) of NH_4^+
**4705**	**4716**	$\nu_3 + \nu_4$ (NH_4^+)	Combination band (Asymmetric stretch + out-of-plane bend) of NH_4^+
4500	-	ν (OH) + δ (Al-Fe-OH)	Combination band of structural OH stretch and bending mode of Al-Fe-OH
3618	3636	ν (OH)	Structural OH stretch
3469	-	ν (H_2O)	H_2O stretch
**3263**	**3279**	ν_3 (NH_4^+)	Asymmetric stretch of NH_4^+
**3070**	**3077**	$\nu_2 + \nu_4$ (NH_4^+)	Combination band (In-plane bend out-of-plane bend) of NH_4^+
1843	-		Combination band or overtone of Metal cation-OH vibrations
1643	1635	δ (H_2O)	Bending vibration of H_2O
**1430**	**1438**	ν_4 (NH_4^+)	Out-of-plane bend of NH_4^+
1132	1176	ν (M^+-O)	Stretching vibration of Metal cation-O group
960	1086	ν (Si-O)	Stretching vibration of Si-O group
835	-	δ (Al-Fe-OH)	Bending vibration of Al-Fe-OH group

Natural montmorillonite

Wavenumber (cm^{-1})	Wavenumber literature (cm^{-1})[4]	Vibrational Assignments	Carrier
5221	-	ν + δ (H_2O)	Combination band of H_2O
4506	-	ν (OH) + δ (Al-Fe-OH)	Combination band of structural OH stretch and bending mode of Al-Fe-OH
3621	3636	ν (OH)	Structural OH stretch
3474	-	ν (H_2O)	H_2O stretch
1841			Combination band or overtone of Metal cation-OH vibrations
1638	1635	δ (H_2O)	Bending vibration of H_2O
1127	1250	ν (M^+-O)	Stretching vibration of Metal cation-O group
1003	1098		Stretching vibration of Si-O group
821	-	δ (Al-Fe-OH)	Bending vibration of Al-Fe-OH group

Supplementary Table 5. Infrared absorption features of ammonia treated and untreated commercial montmorillonite samples measured at 300 K and 1 atm pressure. Spectra of the samples are provided in Figure 1g of the main article.

NH_3 -Commercial montmorillonite

Wavenumber (cm^{-1})	Wavenumber literature (cm^{-1})[6,8]	Vibrational Assignments	Carrier
5230	-	$\nu + \delta$ (H_2O)	Combination band of H_2O
4534	-	ν (OH) + δ (Al-Fe-OH)	Combination band of structural OH stretch and bending mode of Al-Fe-OH
3621		ν (OH)	Structural OH stretch
3458	3430	ν (H_2O)	H_2O stretch
**3270**	**3278**	ν_3 (NH_4^+)	Asymmetric stretch of NH_4^+
**3090**	**3086**	$\nu_2 + \nu_4$ (NH_4^+)	Combination band (In-plane bend and out-of-plane bend) of NH_4^+
1847		-	Combination band or overtone of Metal cation-OH vibrations
1637	1635	δ (H_2O)	Bending vibration of H_2O
**1430**	**1430**	ν_4 (NH_4^+)	Out-of-plane bend of NH_4^+
1198	-	ν (M^+-O)	Stretching vibration of Metal cation-O group
932	-	ν (Si-O)	Stretching vibration of Si-O group
840	-	δ (Al-Mg-OH)	Stretching vibration of Al-Mg-OH group

Commercial montmorillonite

Wavenumber (cm^{-1})	Wavenumber literature (cm^{-1})[8,9]	Vibrational Assignments	Carrier
5160	5250	ν + δ (H_2O)	Combination band of H_2O
4543	4535	ν (OH) + δ (Al-Al-OH)	Combination band of structural OH stretch and bending mode of Al-Al-OH
3614	3632	ν (OH)	Structural OH stretch
1625	1635	δ (H_2O)	Bending vibration of H_2O
1031	1040	ν (Si-O)	Stretching vibration of Si-O group
832	840	δ (Al-Mg-OH)	Stretching vibration of Al-Mg-OH group
556	523		Bending vibrations of Si-O-Si bond

Supplementary Notes

Supplementary Note 1: Ammonia ice calibration

Ammonia gas was deposited at 2×10^{-8} torr on the cold (5 K) silver mirror through a glass capillary to form ammonia-ice. The thickness of the ice was determined *online* via laser interferometry. A He-Ne laser (λ = 632.8 nm) is reflected off the silver substrate at an incident angle (θ_i) of 4° to obtain an interferogram. Supplementary Fig. S3 shows an interferogram measured during ammonia NH_3 ice deposition, the number of interference fringes (m) observed in the spectrum is equal to 2.5. The thickness (d) of ammonia ice is determined using equation (1)

$$d = \frac{m\lambda}{2\sqrt{n^2 - \sin^2\theta}} \qquad (1)$$

where, n is the refractive index of ammonia (n = 1.38)[10].

Supplementary Note 2: Column density calculation

Column density (N) of NH_4^+ ions and interlayer water molecules were determined at 1420 (ν_4) and 1637 (δ) cm^{-1} respectively, using a modified Lambert-Beer law equation given as:

$$N = \frac{\ln 10 \int_{\nu 1}^{\nu 2} A_\nu d\nu}{A_{exp}} \frac{\cos(\theta = 45^0)}{2}$$

where $\int_{\nu 1}^{\nu 2} A_\nu d\nu$ is the integral peak area of the absorbance in the region ν_1-ν_2 cm^{-1}, A_{exp} is the integrated absorption coefficient in units of cm $molecule^{-1}$, θ is the angle of incident IR beam from the normal of the substrate. The integrated absorption coefficients (A_{exp}) of NH_4^+ ν_4 bending mode (1.50×10^{-16} cm $molecule^{-1}$) and H_2O deformation band (1.1×10^{-17} cm $molecule^{-1}$) were determined from the literature.[11,12]

The absorbance (A_ν) of the peak at 1420 cm^{-1} (ν_4 mode of NH_4^+) was recorded in-situ during the TPD at every 2 K rise in the temperature. Column density was derived for each absorbance values using the abovementioned equation and then plotted against temperature. The onset of the band at 1420 cm^{-1} i.e. column density > 0, is observed at 54 ± 6 K. Indicating that the acid-base chemistry leading to the formation of ammonium ion could occur at very low temperature.

Supplementary References

1 Jones, B. M. & Kaiser, R. I. Application of Reflectron Time-of-Flight Mass Spectroscopy in the Analysis of Astrophysically Relevant Ices Exposed to Ionization Radiation: Methane (CH_4) and D4-Methane (CD_4) as a Case Study. *J. Phys. Chem. Lett.* **4**, 1965-1971, (2013).

2 Bennett, C. J. *et al.* High-Sensitivity Raman Spectrometer To Study Pristine and Irradiated Interstellar Ice Analogs. *Anal. Chem.* **85**, 5659-5665, (2013).

3 Maksyutenko, P., Muzangwa, L. G., Jones, B. M. & Kaiser, R. I. Lyman α photolysis of solid nitromethane (CH_3NO_2) and D3-nitromethane (CD_3NO_2) – untangling the reaction mechanisms involved in the decomposition of model energetic materials. *Phys. Chem. Chem. Phys.* **17**, 7514-7527, (2015).

4 Ferrari, M. *et al.* Reflectance spectroscopy of ammonium-bearing phyllosilicates. *Icarus* **321**, 522-530, (2019).

5 Moore, M. H., Ferrante, R. F., Hudson, R. L. & Stone, J. N. Ammonia–water ice laboratory studies relevant to outer Solar System surfaces. *Icarus* **190**, 260-273, (2007).

6 Berg, B. L. *et al.* Reflectance spectroscopy (0.35–8µm) of ammonium-bearing minerals and qualitative comparison to Ceres-like asteroids. *Icarus* **265**, 218-237, (2016).

7 Ehlmann, B. L. *et al.* Ambient and cold-temperature infrared spectra and XRD patterns of ammoniated phyllosilicates and carbonaceous chondrite meteorites relevant to Ceres and other solar system bodies. *Meteorit. Planet. Sci.* **53**, 1884-1901, (2018).

8 Bishop, J. L., Banin, A., Mancinelli, R. L. & Klovstad, M. R. Detection of soluble and fixed NH_4^+ in clay minerals by DTA and IR reflectance spectroscopy: a potential tool for planetary surface exploration. *Planet. Space Sci.* **50**, 11-19, (2002).

9 Bishop, J. L., Lane, M. D., Dyar, M. D. & Brown, A. J. Reflectance and emission spectroscopy study of four groups of phyllosilicates: smectites, kaolinite-serpentines, chlorites and micas. *Clay Minerals* **43**, 35-54, (2018).

10 Satorre, M. Á., Leliwa-Kopystynski, J., Santonja, C. & Luna, R. Refractive index and density of ammonia ice at different temperatures of deposition. *Icarus* **225**, 703-708, (2013).

11 Pankewitz, T., Lagutschenkov, A., Niedner-Schatteburg, G., Xantheas, S. S. & Lee, Y.-T. Infrared spectrum of $NH_4^+(H_2O)$: Evidence for mode specific fragmentation. *J. Chem. Phys.* **126**, 074307, (2007).

12 Bouilloud, M. *et al.* Bibliographic review and new measurements of the infrared band strengths of pure molecules at 25 K: H_2O, CO_2, CO, CH_4, NH_3, CH_3OH, HCOOH and H_2CO. *Mon. Not. R. Astron. Soc.* **451**, 2145-2160, (2015).
